# Model yeast as a versatile tool to examine the antioxidant and anti-ageing potential of flavonoids, extracted from medicinal plants

**DOI:** 10.3389/fphar.2022.980066

**Published:** 2022-09-02

**Authors:** Hira Zahoor, Kwanrutai Watchaputi, Janejira Hata, Wachirachai Pabuprapap, Apichart Suksamrarn, Lee Suan Chua, Nitnipa Soontorngun

**Affiliations:** ^1^ Gene Technology Laboratory, Division of Biochemical Technology, School of Bioresources and Technology, King Mongkut’s University of Technology Thonburi, Bangkok, Thailand; ^2^ Department of Chemistry and Center of Excellence for Innovation in Chemistry, Faculty of Sciences, Ramkhamhaeng University, Bangkok, Thailand; ^3^ Metabolites Profiling Laboratory, Institute of Bioproduct Development, Universiti Teknologi Malaysia, Johor Bahru, Johor, Malaysia

**Keywords:** flavonoids, quercetin analogues, model yeast, oxidative stress response, anti-ageing, medicinal plant, morin, antioxidant

## Abstract

The demand for the production of herbal extracts for cosmetics, food, and health supplements, known as plant-based medicine, is rising globally. Incorporating herbal extracts could help to create higher value products due to the functional properties of bioactive compounds. Because the phytochemical composition could vary depending on the processing methods, a simple bioassay of herbal bioactive compounds is an important screening method for the purposes of functional characterization and quality assurance. As a simplified eukaryotic model, yeast serves as a versatile tool to examine functional property of bioactive compounds and to gain better understanding of fundamental cellular processes, because they share similarities with the processes in humans. In fact, aging is a well-conserved phenomenon between yeast and humans, making yeast a powerful genetic tool to examine functional properties of key compounds obtained from plant extracts. This study aimed to apply a well-established model yeast, *Saccharomyces cerevisiae*, to examine the antioxidant and anti-aging potential of flavonoids, extracted from medicinal plants, and to gain insight into yeast cell adaptation to oxidative stress. Some natural quercetin analogs, including morin, kaempferol, aromadendrin, and steppogenin, protected yeast cells against oxidative stress induced by acetic acid, as shown by decreased cell sensitivity. There was also a reduction in intracellular reactive oxygen species following acetic acid treatment. Using the chronological aging assay, quercetin, morin, and steppogenin could extend the lifespan of wild-type *S. cerevisiae* by 15%–25%. Consistent with the fact that oxidative stress is a key factor to aging, acetic acid resistance was associated with increased gene expression of *TOR1*, which encodes a key growth signaling kinase, and *MSN2* and *MSN4*, which encode stress-responsive transcription factors. The addition of the antioxidant morin could counteract this increased expression, suggesting a possible modulatory role in cell signaling and the stress response of yeast. Therefore, yeast represents a versatile model organism and rapid screening tool to discover potentially rejuvenescent molecules with anti-aging and anti-oxidant potential from natural resources and to advance knowledge in the molecular study of stress and aging.

## Introduction

The concept of preparation of medicinal plant for experimental purposes involves the proper and timely collection of the plants, authentication by an expert, adequate drying, and grinding, followed by extraction, fractionation, and/or isolation of the bioactive compound where applicable (Abubakar and Haque, 2020). In addition, it comprises determination of quantity and quality of bioactive compounds using either *in vitro* or *in vivo* assays. The medicinal plants are historically used and continually gaining more international popularity as a source of medicinal or pharmacological ingredients in product formulation because of its natural origin, availability in local communities, cheap to purchase, and ease of administration (Wachtel-Galor et al., 2011). Also, herbal medicine may be useful alternative treatment in case of numerous diseases and disorders (Sen and Samanta, 2015).

Extraction of medicinal plants is a process of separating active plant materials or secondary metabolites such as alkaloids, flavonoids, terpenes, saponins, steroids, and glycosides from inert or inactive material using an appropriate solvent and standard extraction procedure (Abubakar and Haque, 2020) Plant materials with high content of phenolic compounds and flavonoids are found to possess antioxidant properties, and hence are used to treat age-related diseases such as Alzheimer’s disease, Parkinsonism, anxiety, and depression (Azwanida, 2015).

Since 2005, the number of old citizens has steadily risen to 11.23 million or 17.13 percent of the total population (Kudryavtseva et al., 2016). Rising awareness regarding the side-effects of synthetic compounds and health benefits offered by phytomedicines and herbal extracts drive the market growth for plant extracts during the ageing era. According to Markets and Markets analysis, the plant extracts market is estimated to be valued at USD 23.7 billion in 2019 and is projected to reach USD 59.4 billion by 2025, at a CAGR of 16.5% from 2019 to 2025. Ageing is broadly defined as a time-sensitive decreasing effectiveness of living organisms. Age is also a key and relevant determinant of cell state, ranging in yeasts to humans. Many characteristics resembling those of humans make yeast a strong instrumental model to gain a better understanding of fundamental phenomena, including ageing (Chadwick et al., 2016). Many studies have widely supported a free radical theory which postulates that oxidative damage to macromolecules is a primary cause of ageing (Harman, 1956). The generation of free radicals internally and externally are therefore age-promoting factors (Anand David et al., 2016). High level reactive oxygen species (ROS) activate cellular defense mechanisms which induce the expression of antioxidant proteins that, in turn, eliminate ROSs as self-defense mechanism (Tran and Green, 2019). Deregulated oxidative stress defense systems and altered levels of intracellular ROSs are related to cancers, cardiovascular disease, autoimmune diseases and also ageing. Therefore, determining and identifying signalling proteins in charge of cellular defense against oxidative stress could be vital in disease intervention (Schieber and Chandel, 2014).

 Functional bioactive molecules including antioxidants and food supplements have revolutionised pharmacological and food industries, considerably improving health and well-being. High intake of food rich in antioxidants has been associated with the decline of degenerative and chronic disease progression caused by oxidative stress (Zhang and Tsao, 2016). Supplemented antioxidants delay the formation of toxic oxidation products and rancidity development, thereby maintaining nutritional quality and extending the shelf-life of products (Shahidi and Ambigaipalan, 2015). Plant-derived antioxidants can be used to prevent ageing and ageing-related diseases as well as inflammation due to antioxidant and anti-inflammatory effects (Anand David et al., 2016).

Flavonoids represent a major group of bioactive compounds found in plants and known for defensive antioxidant mechanisms against biotic and abiotic stresses. Naturally flavonoids do not exist in its pure form usually exist together with other compounds a dietary food rich in flavonoids, often comes with other compounds, and flavonoids can also interact with other compounds, such as carbohydrate, fat, protein, acid, etc. Food component interaction is closely related to the change of flavonoid’s own characteristics, and it may change a variety of physiological activities of flavonoids *in vivo*. (Chen et al., 2021).

Previously, the extract of pigmented Hom Dang rice bran that contains high levels of free phenolic acids and flavonoids, including quercetin, has been shown to extend the lifespan of *S. cerevisiae* (Sunthonkun et al., 2019). Quercetin can be found in various fruits and vegetables and is emerging as a strong anti-oxidant flavonoid found in food because of its ability to neutralise free radicals and promote growth (Liguori et al., 2018). Quercetin and its derivatives are important flavonoid that show diverse biological activities, such as antioxidant, anticarcinogenic, anti-inflammatory and antiviral activities. Although quercetin exhibits a wide range of pharmacological properties, its application is still difficult because of its low oral bioavailability, short elimination half-life and low titre, restricting its further development (Wright et al., 2010). Different substituents in the flavonoid skeleton of quercetin may change the biochemical activity and bioavailability of analogue molecules and enhance their biological properties.

Since *S. cerevisiae* is an excellent model organism, it is often used to investigate the potential effects of bioactive compounds on some highly conserved cellular processes in men (Zimmermann et al., 2018). For example, the molecular mechanism underlying oxidative stress is one process, efficiently analysed using the yeast models. *S. cerevisiae* undergoes a programmed cell death process in response to lethal concentrations of acetic acid at 80 mM or above (Chaves et al., 2021). It is not merely a food preservative but also a byproduct of fermentation and an inducer of oxidative stress, causing yeast cell death and accelerating ageing. Chronological ageing provides strong evidence for the role of acetic acid and acidification as accelerators of yeast ageing process, which may be more relevant to mammalian ageing than expected (Palma et al., 2018). Mammalian tumor cells, maintained in stationary culture, lose viability by lactate media acidification (Fabrizio and Wei, 2011). Therefore, acidification may have a conserved role in accelerating cellular ageing in higher eukaryotes (Mirisola and Longo, 2012). Characterization of cell components and mechanisms involved in yeast acetic acid-induced apoptosis have been reported (Giannattasio et al., 2013). Recently, coordination of stress responses and the roles of different stresses on yeast cell ageing are documented (Dawes and Perrone, 2020). Here, we aimed to demonstrate the use of model yeast *S. cerevisiae* as a rapid screening platform to investigate the potential antioxidant and anti-ageing properties of quercetin analogues, previously found as a key component of various medicinal plant extracts, in promoting lifespan extension and the cell response during the acetic acid-induced oxidative stress. Using this simple *in vivo* model yeast assay will help us to uncover hidden biological properties of natural bioactive compounds and perhaps find their potential uses as supplemented food, cosmetic ingredients or components of healthcare products.

## Materials and methods

### *S. cerevisiae* strains and growth conditions

*S. cerevisiae* wild-type and mutant strains BY4742 (WT) and single-deletion strains were obtained from Open Bio System (Dharmacon, Inc., Lafayette, CO, United States). Yeast wild-type (BY4742) (WT) strains was routinely grown in Yeast Extract-Peptone-Dextrose (YPD) medium and incubated at 160 rpm and 30°C. For chronological studies, yeast cells were inoculated in minimal medium containing a yeast nitrogen base. Dimethyl sulfoxide (DMSO) and 2′,7′-dichlorofluorescin diacetate (H_2_DCFDA) were purchased (Aldrich-Sigma, United States).

### Isolation of flavonoids from plants

The flavonoids quercetin and 3,5,7,4′-tetrahydroxy-2′-methoxyflavone were isolated from *Anaxagorea luzonensis* A. Gray, whereas morin, kaempferol, 2,3-trans-dihydromorin, (+)-aromadendrin and steppogenin were isolated from *Maclura cochinchinensis* (Lour.) Corner. Briefly, the pulverized, dried heartwood of *A. luzonensis* (1.0 kg) was macerated successively with n-hexane, EtOAc and MeOH to yield, after evaporation of the solvents under reduced pressure, the n-hexane (11.0 g), EtOAc (99.5 g), and MeOH (43.0 g) extracts, respectively. The EtOAc extract was fractionated on a silica column, using a gradient solvent system of n-hexane–EtOAc, EtOAc and EtOAc–MeOH with increasing amounts of the more polar solvent. The eluates were examined by TLC, and ten combined fractions were obtained. Fraction 10 was column chromatographed, using CH_2_Cl_2_ and CH_2_Cl_2_–MeOH with increasing amounts of the more polar solvent to give two subfractions. The second subfraction, upon standing, a yellow solid separated out, which was rechromatographed eluting under isocratic condition of 6% MeOH in CH_2_Cl_2_ to yield quercetin (350 mg) as a pale yellowish powder. The crude MeOH extract (40.0 g) was fractionated by silica column chromatography, using a gradient solvent system of EtOAc and EtOAc–MeOH with increasing amount of the more polar solvent. The eluates were examined by TLC and five combined fractions were obtained. Fraction 4 was chromatographed eluting with CH_2_Cl_2_–EtOAc (65:35) to yield 3,5,7,4′-tetrahydroxy-2′-methoxyflavone (5 mg) as a white powder. The NMR spectroscopic data of the two isolated compounds were consistent with the reported values (Gonda et al., 2000; Pabuprapap et al., 2019). The pulverized, dried heartwood of *M. cochinchinensis* (1.0 kg) was extracted successively with n-hexane, EtOAc and MeOH in the same manner to that of *A. luzonensis* to give the hexane (1.24 g), EtOAc (159.2 g) and MeOH (101.8 g) extracts. MeOH–H_2_O (1:1v/v, 100 ml) was added with stirring to the EtOAc extract and the solid that precipitated out was collected by filtration and recrystallized from MeOH–H_2_O (1:1) to yield morin (1.1 g). The evaporated residue was fractionated by silica column chromatography in similar manner to that of *A. luzonensis* to afford eleven combined fractions. Fraction 5 was repeatedly recrystallized with n-hexane–EtOAc (100:70) to give kaempferol (210 mg). Fraction 6 upon standing impure solid separated out, which was chromatographed on Sephadex LH-20 eluting with MeOH to yield steppogenin (53 mg). Fraction 8 was crystallized from n-hexane–EtOAc (100:75) to give (+)-aromadendrin (30 mg). Fraction 10 was chromatographed using CH_2_Cl_2_–MeOH (100:6) to afford 9 subfractions. Subfraction 7 was chromatographed over silica gel using CH_2_Cl_2_–MeOH (100:6) to give 6 subfractions. Subfraction 2 was chromatographed on Sephadex LH-20 eluting with MeOH to yield 2,3-trans-dihydromorin (20 mg). The NMR spectroscopic data of the five isolated compounds were consistent with the reported values (Sharififar et al., 2003; Jeong et al., 2009; Jeon et al., 2011; Zheng et al., 2011; Wahab and Begum, 2014).

### Protective effect of quercetin and analogues during acetic acid stress

For the pretreatment with quercetin analogues, WT BY4742 was inoculated into YPD overnight at 150 rpm and 30°C. Cells were regrown in YPD until an OD_600_ of 0.6 and pretreated with 200 µM of analogues or quercetin. Subsequently, the cells were transferred into a 96-well plate, and cell dilutions were made and incubated in a shaking incubator at 150 rpm and 30°C. After 24 h, the cells were incubated with 50 mM of AA under shaking at 150 rpm and 30°C, and the OD_600_ was determined after 24 h.

### Assessment of anti-ageing effects of quercetin and analogues on yeast cells *via* the chronological life-span assay

Wild-type cells were grown in Yeast Peptone dextrose (YPD) media in the presence or absence of extract or compounds; assays were carried out in Yeast nitrogen based (YNB) media with amino acids. Cultures were inoculated into YPD medium at a flask volume/medium volume ration of 5:1. Subsequently, quercetin or analogues at a final concentration of 200 µM was added and incubated at 30°C with shaking at 160 rpm. From day 1 of the CLS assay, aliquots were sampled at day 1, 3, 5, 7, 9, 10, 12, 14, 16, 18, 20 up to day 35 for analysis. Cellular viability was examined by the methylene blue method using Thomas’s counting chamber. The numbers of stained (non-active) or un-stained (active) cells is used for the determination % of viability. Were the percentages of “alive” cells (%MB “viability”), which is the number of nonpigmented cells (live cells) divided by the total number of cells (stained and unstained), determined as described previously (Sunthonkun et al., 2019).

### Dichloro-dihydro-fluorescein diacetate assay for reactive oxygen species detection

After overnight culturing of wild-type cells in YPD, cells were regrown in YNB medium. Upon reaching an OD_600_ of 0.6, they were pretreated with 200 µM quercetin or analogues and incubated in a shaking incubator at 150 rpm and 30°C. After 24 h of incubation, cells were treated with acetic acid or hydrogen peroxide for 10 min, followed by washing with PBS buffer and dyeing with DCFH-DA dye for 30 min at 150 rpm and 30°C. Cells were harvested and washed with PBS buffer twice and subsequently lysed using lysis buffer and glass beads, followed by vortexing three times with a 2-min gap in between on ice. Cells were then centrifuged on ice, and the supernatant was collected in a 96-well plate and observed under a fluorescent microplate reader. The protein concentration was measured using a Nano Drop spectrophotometer (Sunthonkun et al., 2019).

### Gene induction and quantitative real-time polymerase chain reaction

The *S. cerevisiae* wild-type strain BY4742 was cultured in YPD at 30°C with shaking overnight. The yeast cells were measured and adjusted at an optical density (OD_600_) of 0.1 for the starter cell. The culture was incubated until obtaining an OD_600_ of about 0.5–0.6. Then, cells were pretreated with Morin prior to be exposed to 50 mM acetic acid or treated with 4 mM of hydrogen peroxide for 1 h. Total RNAs were extracted as described by (Schmitt et al., 1990). RNA was dissolved by DEPC and purified using the RNeasy Mini Kit (QIAGEN, Hilden, Germany). The purified RNA was used to synthesize cDNA by using the qPCRBIO cDNA synthesis kit (PCRBIOSYSTEMS, United Kingdom). The qRT-PCR assays were performed using a Real-Time PCR Detection System with a software for analysis. The reaction mixtures contained Universal qPCR Master Mix (NEB). Gene-specific oligonucleotides were used.

The primers used were:

*MSN2*: 5′-ACC​GTC​ACC​TTC​ATC​GGT​A and 5′-CGC​TAA​ATC​TTC​GGC​GTG​A.

*MSN4*:5′- CCT​TCT​TGA​GGC​AGA​ACC​TTC and 5′-CTG​TAT​CTT​CTT​GCG​TCG​CG.

*ASG1*: 5′-AAT​AGC​GCC​TCC​AGC​AAC and 5′-CAC​GTT​GAT​AAA​CGT​GCG​G.

*TOR1*: 5′-GCA GCC TCA TCT GGTTACG and 5′-GAG ACA TGC CCT GCATGAG.

*SCH9*: 5′-AGC​AGC​TGC​TTA​TGG​TCC and 5′-ATG​ATG​CTG​GCT​AGC​AGC.

*RIM14*: 5′-GCG​ATA​TGG​CTC​TCC​ACA and 5′-GCC​TGC​GCT​ACC​TTC​ATA.

*SIR2*: 5′-CAC​ACT​AAA​GCT​GCG​CTC and 5′-CAT​TCG​AGC​ATT​GAG​AGA​CTC​TC.

*GPX1*: 5′-AGT​GAT​TGT​GGC​CTT​TCC and 5′-CAG​ACT​TCC​CGC​TTA​CTG​A.

*GPX2*: 5′-CCC​ATG​TAA​TCA​TTC​GGG and 5′GGA​CAA​CCT​TAC​CAT​TGG.

*SOD1*: 5′-CAA​GAA​GAC​ACA​TGG​TGC and 5′-CGG​AGG​TAG​GAC​CGA​TAA.

*SOD2*: 5′-TCT​CAG​ATC​TTC​TGG​CCA​AG and 5′-GTC​GAT​TGC​CTT​TGC​CAA.

The relative expression data were analyzed using the 2^–ΔΔCt^ method.

### Statistical analysis

Data are presented as means ± SEM (*n* = 6; *p < 0.05; the *p* values for comparing the means of two groups were calculated using the IBM SPSS statistics software using *t*-test (IBM Corp., Armonk, NY). Also, data are presented as means ± SEM (*n* = 6; *p < 0.05; the *p* values for comparing.

## Results

### Pretreatment with quercetin analogues protected cells from acetic acid stress

**FIGURE 1:**
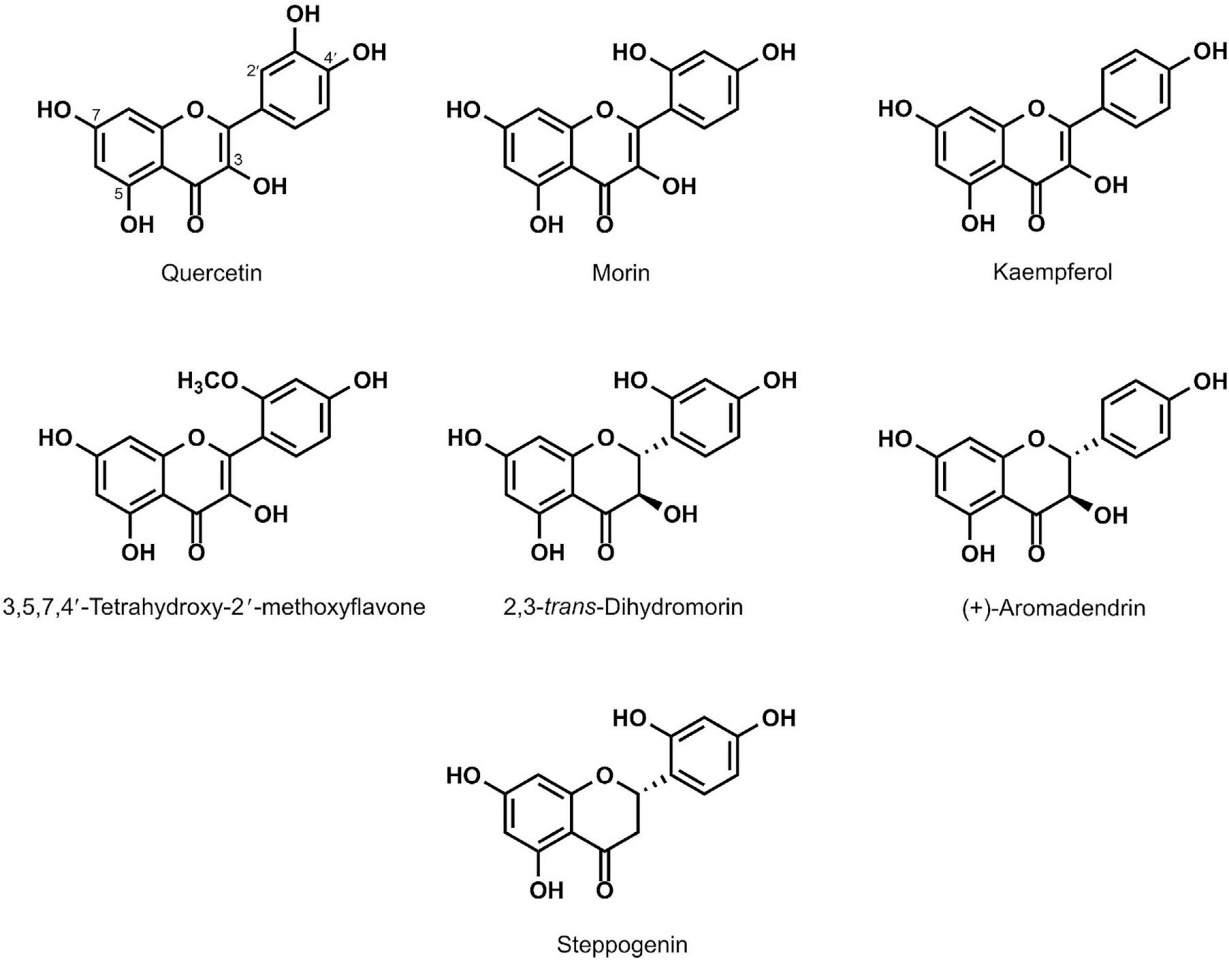
Structures of quercetin and analogues used in this study.

**FIGURE 2:**
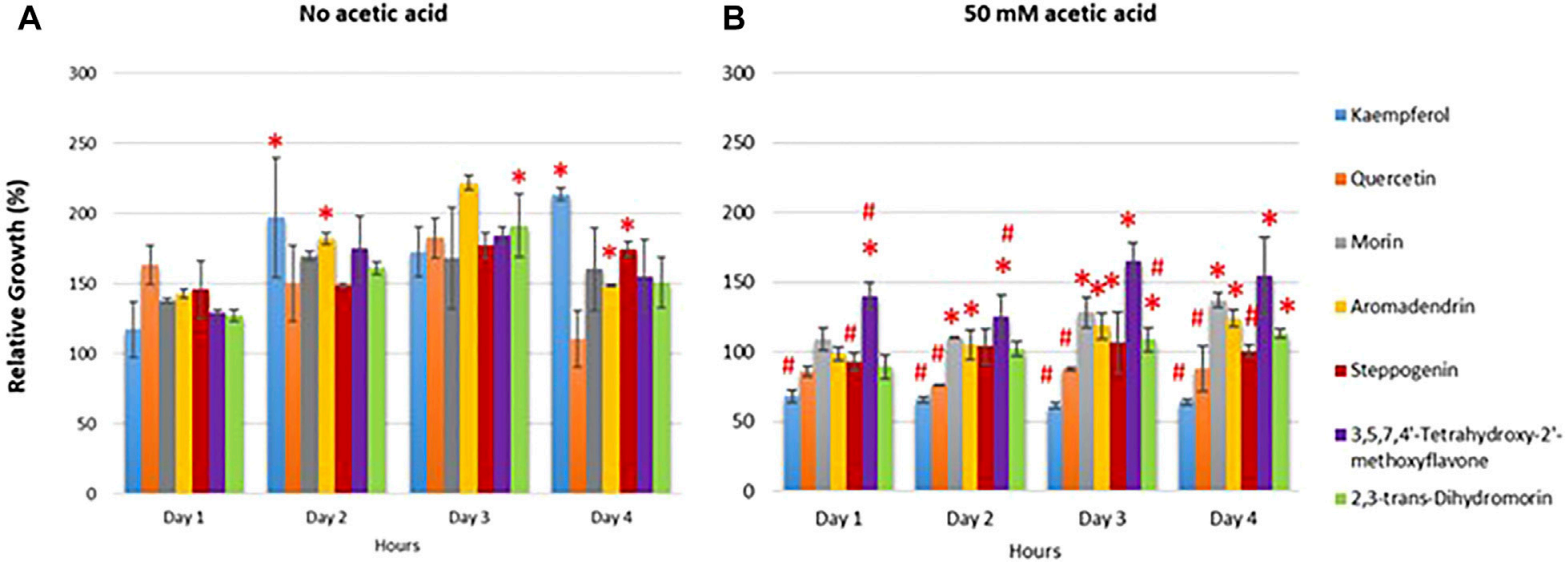
Sensitivity of wild-type *S. cerevisiae* strain treated with selected flavonoids. Cells were pretreated with compounds as indicated and the optical cell density (OD_600_) was obtained at day1–4. The relative growth (%) was measured in the absence or presence of tested compounds using the solvent DMSO. The growth curves were first obtained and the relative growth was expressed as normalized to untreated cells under conditions **(A)** without acetic acid **(B)** treated with 50 mM acetic acid. Detail of quercetin analogues is provided in Figure 1. At least two independent experiments were performed in triplicates with *p value < 0.05 and #*p* value < 0.01compared to quercetin-treated or morin-treated cells, respectively.

Plants contain bioactive compounds with promising biological activities to reduce toxic radicals. These include well-known antioxidants namely resveratrol and quercetin with promising health promoting benefits (Dhalaria et al., 2020). Most extensive research on antioxidants from natural products is on flavonoids. In this study, the flavonoids quercetin and 3,5,7,4′-tetrahydroxy-2′-methoxyflavone were isolated from the plant *Anaxagorea luzonensis* A. Gray, a species of tree in the family Annonaceae. The morin, kaempferol, 2,3-trans-dihydromorin, (+)-aromadendrin and steppogenin were isolated from *Maclura cochinchinensis* (Lour.), commonly known as cockspur thorn, is a species of vine or scrambling shrub in the family Moraceae. To examine the antioxidant potential of flavonoids (Figure 1), yeast cells were treated with a sub-lethal concentration of 50 mM acetic acid and examined the protective effects of different natural flavonoids namely quercetin, kaempferol and morin, 3,5,7,4′-tetrahydroxy-2′-methoxyflavone which are the flavone-type of flavonoids, and 2,3-trans-dihydromorin, aromadendrin and steppogenin, which are flavanone-type of flavonoids, on cell growth in the wild-type (WT) *S. cerevisiae* BY4742 strain. First, these flavonoids promoted the growth of *S. cerevisae* under normal physiological condition (no acetic acid). As shown, kaempferol, morin, aromadendrin and steppogenin showed similar or slightly better growth promoting effect as compared to quercetin (Figure 2A) (Kumar and Pandey, 2013). These flavonoids also protected yeast cells from acetic acid-induced cellular toxicity (Figure 2B). Exposure to acetic acid increased sensitivity of yeast cells as compared to the untreated condition (Figure 2B).

### Structure-activity relationship of flavonoids

The antioxidant property of flavonoids derives from their scavenging activity (Banjarnahor and Artanti, 2015). Here, growth of yeast cells in the presence of potent oxidant and ROS generator acetic acid was first examined to investigate the antioxidant potential of quercetin and some analogues. To eliminate the reactive oxygen species, they could donate a hydrogen atom or transfer the single-electron to chelate free radicals. Here, *in vivo* investigation was carried out using a yeast model to examine the correlations between structural feature of flavonoids and their antioxidant activities. As compared to quercetin, kaempferol, a tetrahydroxyflavone lacking 3′-hydroxy site on the B ring, displayed the lowest antioxidant potential as shown by the lowest cell growth in the presence of acetic acid (Figure 2B). While morin, a pentahydroxyflavone bearing three hydroxyl substituents at positions 2′, 4′ and 5 showed the best growth, suggesting good antioxidant potential against acetic acid stress (Figure 2B). Despite, the lack of 3′-hydroxy site on the B ring, morin contains a 2′-hydroxy site and equivalent numbers of hydroxyl groups as quercetin, in agreement with their comparable antioxidant potential. Then, another morin analogue 2,3-trans-dihydromorin, lacking the C2-C3 unsaturated bond combined with the C-4 carbonyl group in the C skeleton ring was used and its antioxidant potential was dropped as compared to morin despite having all five hydroxyl groups (Figure 2B). The results suggested that resonance capability of C ring is also important. As shown, quercetin, morin and kaempferol also contained this type of skeleton of flavonol or 3-hydroxyflavone thus different antioxidant activity was due to the presence of hydroxyl groups in the B ring. Next, (+)-aromadendrin or known as the dihydrokaempferol showed comparable antioxidant potential as quercetin and morin and appeared to have better antioxidant activity than kaempferol (Figure 2B), suggesting the importance of hydroxyl groups in the B ring for suppression of ROS effect (Bubols et al., 2013).

Steppogenin which is a 2-(2,4-dihydroxyphenyl)-5,7-dihydroxy-2,3-dihydrochromen-4-one, lacking the 4′ 3-hydroxyl group but having the 2′-hydroxyl group displayed moderate antioxidant potential when compared to (+)-aromadendrin, confirmed that the 3-hydroxyl group of C ring is also important although through a lesser extent as compared to the hydroxy groups in the B ring. Lastly, 3,5,7,4′-tetrahydroxy-2′-methoxyflavone in which the free hydroxyl group at the 2′-position is methylated further increased the antioxidant potential as compared to morin with the 2′-hydroxyl group or more obviously to kaempferol with a hydrogen bond at this position. A single difference among these analogues at the 2′- position of the B ring suggested that this position also contributes to antioxidant activity. The 3,5,7,4′-tetrahydroxy-2′-methoxyflavone appeared to be the most effective compound tested. In agreement, previous work has shown that the antioxidant capacity within flavonoids is also due to the differences in the hydrophobicity and molecular planarity. This is exemplified by the methylated flavones with better inhibitory activity, metabolically steady and intestinal absorption as compared to the unmethylated analogues (Wen and Walle, 2006; Walle et al., 2007).

To conclude, the structure of flavonoids greatly affects their antioxidant activity, namely the presence of hydroxyl groups, the 2,3-unsaturated bond combined with a 4-carbonyl group in the C skeleton, the conjugation between the A and B rings of flavonoids. These would allocate a resonance effect of the aromatic nucleus which generates a stable flavonoid radical, critical for their antioxidant potential during the acetic acid stress. Thus, the model yeast could be effectively and rapidly used to investigate structure-antioxidant activity relationship of flavonoids and other bioactive compounds. Among, the natural analogues of quercetin, morin displayed very good antioxidant potential, it was then selected to examine ability to reduce accumulation of free radicals in yeast cells. Based on the recovery of growth in the presence of these bioactive analogues, the effects observed are further investigated whether they are directly proportional to the antioxidant potential of the compounds via ROS assay.

### Intracellular reactive oxygen species detection for quercetin analogue-treated cells during acetic acid stress

**FIGURE 3:**
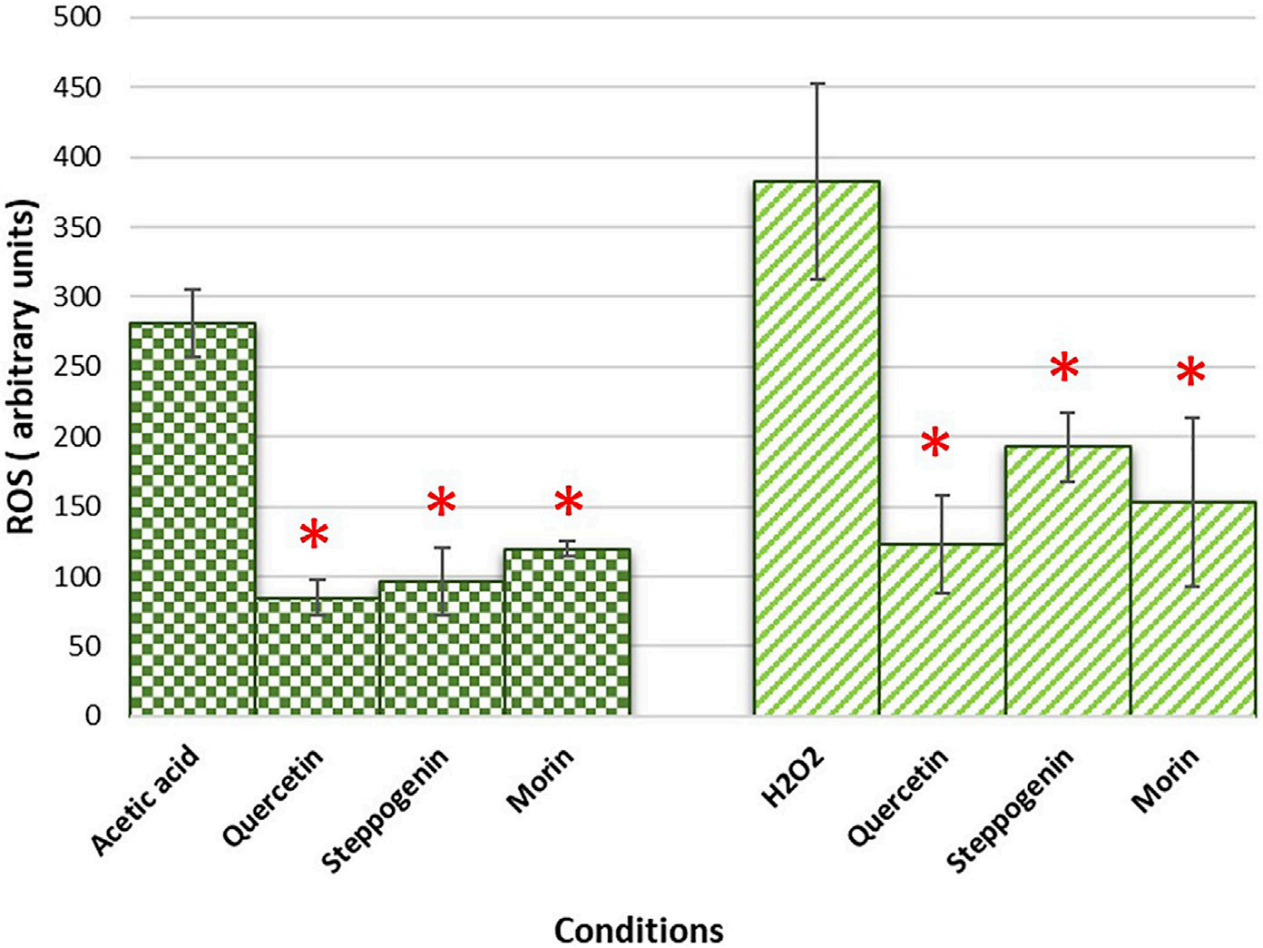
Quantification of intracellular reactive oxygen species accumulated during treatment with acetic acid or H_2_O_2_ in the wild-type *S. cerevisiae* strain pretreated with quercetin, steppogenin or morin after 10 min of treatment with the indicated oxidant. At least two independent experiments were performed in triplicates with #*p* value < 0.05 when compared to DMSO-treated cells.

Intercellular reactive oxygen species (ROSs) is the main component to promote ageing. They destroy several cellular component, including cell membrane leading to cell death (Bouzid et al., 2015). Quercetin pretreatment has been shown to reduce the cellular ROS accumulation, leading to increased percent viability of yeast cells from the hydrogen peroxide treatment (Belinha et al., 2007). To examine their protection against oxidative stress, the wild-type *S. cerevisiae* strain was pre-treated or untreated with morin, quercetin (an analogue of morin with the ortho-dihydroxy arrangement in the B ring) or steppogenin (an analogue lacking the 2,3-unsaturated bond). Cells were first pretreated with these selected antioxidants prior to be exposed to the sub-lethal concentrations of 50 mM acetic acid or 5 mM of hydrogen peroxide for 1 h or 10 min, respectively. The ROSs were tracked with fluorescent DCFH-DA dye to determine ROS levels. The results indicated that under acetic acid or hydrogen peroxide stress, after normalized with the no oxidant condition, cells contain elevated intracellular ROS levels (Figure 3). As compared to the analogue-pretreated cells, the pretreatment could significantly reduce accumulation of intracellular ROS levels by at least 2 to 3-fold as compared to non-pretreated cells (Figure 3), suggesting for protection of yeast cells against the oxidant-induced cellular damages. Likewise, pretreatment with morin or quercetin decreased the intracellular ROS to similar levels (Figure 3), suggesting their equivalent ability to reduce oxidative stress or to scavenge intracellular free radicals. Steppogenin showed lower ability to reduce ROS level in the presence of acetic acid as compared to morin or quercetin (Figure 3). This may be due to the absence of the 2,3-unsaturated bond with a 4-carbonyl group and the 3-hydroxyl group in the C skeleton, thereby reducing its ability to donate hydrogen atom to neutralize the hydroxyl radicels. Thus, the number of hydroxyl groups dominate the antioxidant activity as compared to the position on the ring structure. However, the hydrogen atom donation ability may not as critical for antioxidants during the acetic acid stress with increased levels of intracellular hydrogen ions.

### Quercetin analogues extended the chronological lifespan of yeast cells

Yeast ageing shares similar features with mammalian post-mitotic ageing (Harwood et al., 2007), and protein oxidation and aggregation of damaged proteins in *S. cerevisiae* and the human central nervous system are highly similar (Teichert et al., 1989). The chronological ageing in budding yeast is heavily associated with ROS accumulation and programmed cell death (Herker et al., 2004), and ROS-mediated cell death has been linked to serious human pathologies and ageing. In the food industry, acetic acid is used as antimicrobial agent while polyphenols including flavonoids are used as antioxidants to increase the shelf-life of functional food and dietary supplements (Harwood et al., 2007).

**FIGURE 4:**
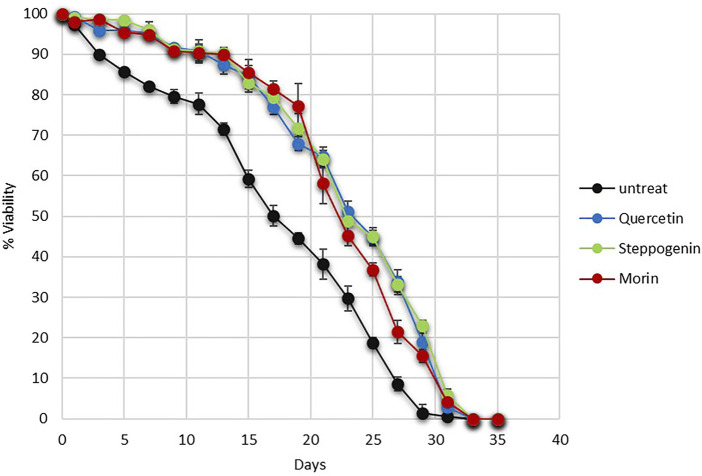
Survival curves of chronologically aging yeast cells from day 0 to day 35 were shown for the wild-type *S. cerevisiae* BY4742. Pretreatment with antioxidants quercetin or steppogenin, or morin. Increasing life-span extension was observed in the wild-type strain as recorded by % MB viability (*p < 0.01, two-tailed Student’s t test compared to untreated condition).

Quercetin not only has pharmacological benefits on health but also delays ageing of many model systems, including yeasts, nematodes and human fibroblast (Alugoju et al., 2018). Here, using yeast as a model of ageing, an ability of morin and steppogenin to extend chronological lifespan (CLS) of the quercetin or analogue-pretreated wild-type (WT) yeast strain was examined. At day 17 of CLS assay, the % viability of the untreated cells was at 50% whereas the quercetin, steppogenin and morin-treated cells were found to be nearly 80% (Figure 4). Finally, the untreated yeast cells died considerably sooner on day 28 as compared to the pretreated cells which live until day 33 or 35 (Figure 4). Examination of structural features of flavonoids including the conserved aromatic structure of the rings and uniform free hydroxyl constituents, there appeared to contribute to their antioxidant activities and anti-ageing efficacy. Thus, flavonoids could serve as a promising pretreatment for oxidative stress and delay of ageing.

### Effect of morin on the enzymatic antioxidant defense system

**FIGURE 5:**
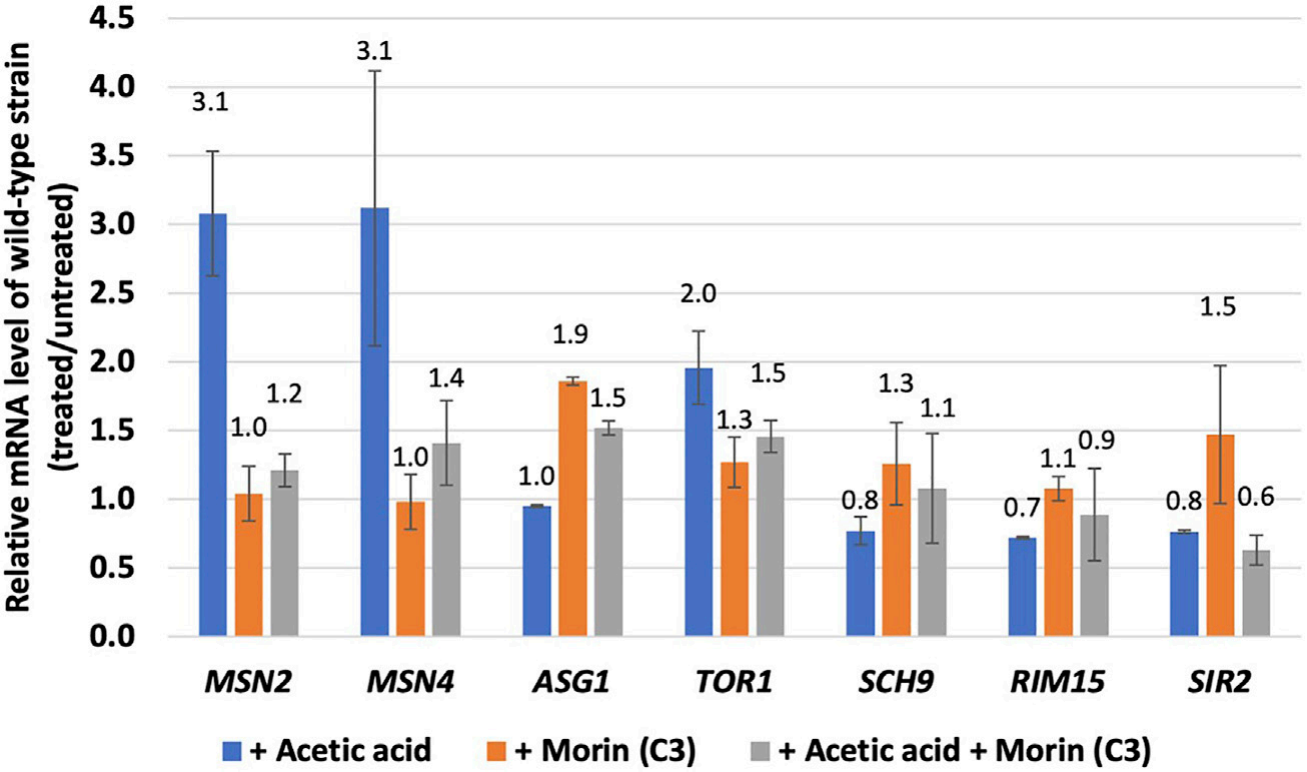
Expression levels of genes involved oxidative stress response and ageing. Relative mRNA levels of wild-type *S. cerevisiae* strain morin-pretreated during the acetic acid stress. At least two independent experiments of qRT-PCR analysis were performed in triplicates.

Next, we questioned how these quercetin analogues might play role in yeast cells at the molecular level. Using gene expression analysis via qRT-PCR, the activation of genes in the Tor1 and Sir2 signalling pathways as well as the transcription factor genes of the stress responses were induced by treatment with morin (C3) as a representative quercetin analogue that has best antioxidant effect and increases the lifespan of *S. cerevisiae* in the CLS assay (Figures 2, 3 and Figure 4A). Pretreatment with morin alone did not alter level of expression of any genes except *ASG1* gene (2-folds) (Figure 4A). However, the acetic acid-induced the expression levels of *TOR1* (2.0-folds), *ASG1* (1.9-fold), and *MSN2* (3.1-fold) were observed but not for *SCH9*, *RIM15* nor *SIR2* (Figure 5A). This finding suggested a co-regulation at *TOR1* gene of the nutrient-signalling pathway and the downstream stress response pathway in response to acetic acid stress (Figure 5A). Interestingly, morin-pretreated cells showed reduced expression of *TOR1*, *MSN2*, and *MSN4* during the acetic-acid exposure (Figure 5A), suggesting its modulatory role in mediating stress signalling and ageing. In *S. cerevisiae* cells, antioxidant defense includes the enzymatic and non-enzymatic processes for scavenging ROSs to reduce the cell toxicity. The lack of cellular defense system results in increase accumulation of toxic radicals and cell death. Overall, our results suggested that morin could modulate cell stress response via two different mechanisms including reduction of ROS accumulation and transcriptional response via repression of Tor1-dependent signalling pathway and down regulation of Msn2/4 function. The latter may be due to its antioxidant property which partially compensates for requirement of antioxidant enzymes to get rid of toxic radicals.

**FIGURE 6:**
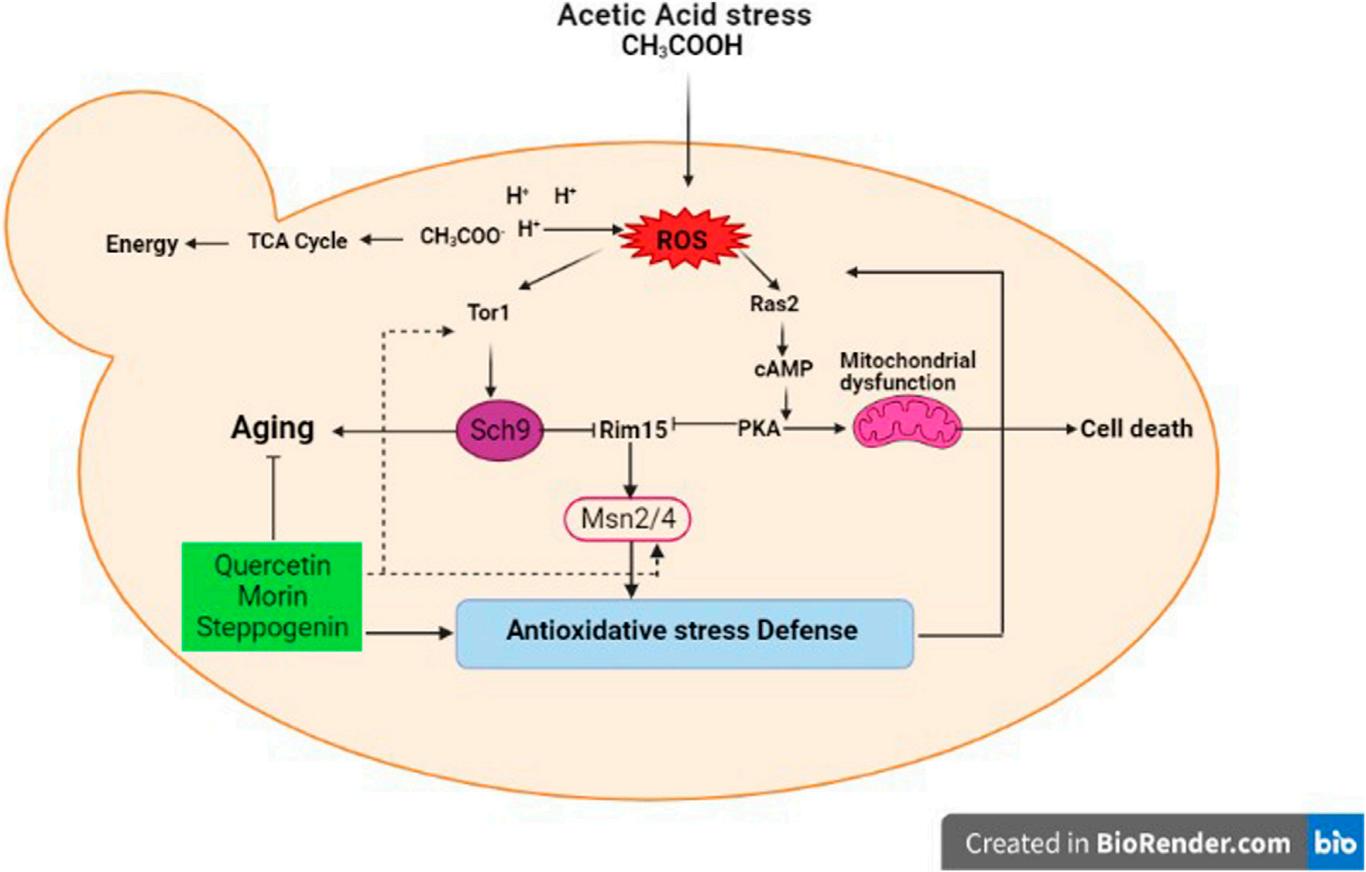
Effect of quercetin, morin and steppogenin treatment in mediating ageing intervention in the model yeast *S. cerevisiae* is proposed. Possible cross-talks between different cellular pathways during the acetic acid stress response under the control of key transcription factors and modulatory signaling kinase of Tor1-dependent pathway are shown.

A model of acetic acid response in *S. cerevisiae* and potential role of some quercetin analogues in mediating ageing intervention and oxidative stress response are proposed (Figure 6). As shown, in yeast, acetic acid also functions as a carbon source by mean of a byproduct of alcoholic fermentation. It has also been shown to have a pro-ageing effect by preventing the entry into a calorie restricted like state (Fabrizio et al., 2004). The imbalance between ROS production and antioxidant defense, leading to oxidative stress and cellular senescence, a physiological mechanism that stops cellular proliferation in response to damages (Liguori et al., 2018). However, the negative effects of free radicals or ROSs are neutralized by antioxidant defense mechanisms (Liguori et al., 2018) either by antioxidants such as quercetin analogues in this case or enzymatic approach. Importantly, the expression of *TOR1* kinase and *MSN2*/*4* stress responsive transcription factor genes are induced in response to acetic acid treatment (Figure 5), indicating that the Tor1-nutrient signaling cascade crosstalk with the cellular stress response pathway. Among others, activation of transcription factors Msn2/4 appears to be modulated by the acetic acid treatment and the presence of antioxidant morin (Figure 5). Many stress-responsive transcription factors are reported to function in concert to modulate proper cellular responses and ensure cell survival (Soontorngun, 2017). Use of enzymatic assays associated with the above kinases, transcription factors and enzymes to elucidate the mechanisms of action of plant biomolecules possessing anti-aging properties will be valuable for product development of anti-aging compounds isolated from plants and other natural products.

## Discussion

Considering that the global population is aging, scientists have aimed to identify new interventions and strategies to delay or ameliorate age-related changes. These include the consumption of “anti-aging” compounds that promote the oxidative stress response and the inactivation of growth signalling. Therefore, there have been growing interests in natural low-molecular-weight antioxidant molecules that could prevent the negative effects of oxidative stress and delay aging: Pretreatment or supplementation with these natural or synthetic analogues of antioxidants could modulate the effect of aging. In fact, from 1940 to 2014, nearly half of the Food and Drug Administration-approved chemical drugs for the treatment of human diseases or disorders were derived from or inspired by natural products (Zhang et al., 2018).

Among others, there is an urgent need to develop effective and selective methods for the extraction, isolation, and functional characterization of bioactive natural compounds. Several methods have been used to extract medicinal plants such as maceration, infusion, decoction, percolation, digestion and Soxhlet extraction, superficial extraction, and ultrasound- and microwave-assisted extraction. Here, flavonoids were isolated carefully from plants by using a series of good practice methods of extraction and fractionation to ensure recovery of phytochemicals. These include choosing the appropriate solvent ratio, extraction conditions, fractionation column, and solvent system, and identifying the eluate via chromatography and spectrophotometry. By incorporating an herbal extract, one can enhance its perceived value and promote the functional claim or biological properties of the products. To produce the desired effect of the product in question, appropriate and well-designed extraction and fractionation techniques are required, and the physiochemical properties of the phytochemicals of interest must be analysed.

In the search for anti-aging interventions, biological screening platforms are important tools for compound and drug discovery. One of the organisms that meets the needs is the budding yeast *S. cerevisiae*, a unicellular fungus that is widely used as a model for human aging and age-related diseases. Its unparalleled genetic tractability combined with the availability of whole genome homozygous and heterozygous gene deletion collections as well as overexpression libraries, make *S. cerevisiae* a versatile toolbox for chemogenomic screens. Due to its short generation time (∼90 min) and modest culturing requirements, yeast can also be grown rapidly in high-throughput experimental setups. In yeast, many pathways that are relevant for aging and disease in humans are well conserved, including nutrient signaling, cell cycle regulation, DNA repair mechanisms, mitochondrial homeostasis, lipostasis, protein folding and secretion, proteostasis, stress response, and regulated cell death (Longo et al., 2012). Indeed, yeast-aging phenotypes are surprisingly similar to human post-mitotic cellular aging. Despite, the determination of antioxidant content of natural plant extracts which contribute to its anti-ageing property can be done either by chromatography methods, such as High-Performance Liquid Chromatography (HPLC) coupled with a Diode-Array-Detector (DAD), Mass Spectroscopy (MS) or fluorescence detector; or using other less specific colorimetric methods, the insight into their biological activities is often lacking through uses of these methods.

In this study, the yeast *S. cerevisiae* has been used as a model organism to screen for functional property of flavonoids. Quercetin-, steppogenin-, or morin-pretreated cells showed increased cell viability during acetic acid stress and chronological aging (Figures 2, 4). Their ability to eliminate reactive oxygen species, generated by high levels of acetic acid is relevant to the structural and functional relationship (Figures 2, 3). These flavonoids contain key features, including conserved aromatic rings and uniform free hydroxyl constituents, that aid in ROS reduction.

Despite the fact that the flavonoids of unmodified forms often shown low bioavailiabity and poor absorption (Chen et al., 2021), with the aids of nano/micro-scale delivery system (Teng et al., 2021), many researchers have reported that a variety of flavonoids can be promising for the development of new drugs, especially to treat neurodegenerative and age-related diseases. This ability is based partly on their ability to reduce ROSs, which provides the rationale for examining the antioxidant and anti-aging potential of flavonoids (de Andrade Teles et al., 2018). Although less is known about steppogenin compared with quercetin, it is a flavonoid that exerts potent anti-neuroinflammatory effects and suppresses the neuroinflammatory responses to lipopolysaccharide (LPS). It inhibits the production of proinflammatory mediators and cytokines in LPS-challenged BV2 and rat primary microglial cells (Kim et al., 2017).

Morin is a super-antioxidant compound, preventing and curing disorders by suppressing ROS (Kataria et al., 2018) and its role in chronic diseases (Sinha et al., 2016). It could be isolated from members of the Moraceae family and extracted from leaves, fruits, stems and branches of numerous plants. Several pieces of evidence suggest that morin could have a beneficial effect on several human diseases. For example, it exerts antioxidant, antidiabetic, anti-inflammatory, antitumoral, antihypertensive, antibacterial, hypouricemic and neuroprotective effects by modulating the activity of many enzymes (Caselli et al., 2016). Morin has been shown to reduce H_2_O_2_-induced intracellular reactive oxygen species generation and nuclear DNA damage. It restores the viability of cells damaged by H_2_O_2_ via inhibition of mitochondrial-dysfunction-mediated apoptosis (Lee et al., 2017). In yeast, acetic acid also functions as a carbon source as a byproduct of alcoholic fermentation. It has also been shown to have a pro-aging effect by preventing entry into a caloric-restriction-like state (Fabrizio et al., 2004) and at high concentrations is antimicrobial. The imbalance between ROS production and antioxidant defense leads to oxidative stress and cellular senescence, a physiological mechanism that stops cellular proliferation in response to damage (Liguori et al., 2018). However, the negative effects of free radicals or ROSs are neutralized by antioxidant defense mechanisms (Liguori et al., 2018), either by antioxidants such as flavonoids or through activation of antioxidant enzymes.

Acetic acid toxicity is related to induction of growth signalling pathways and that oxidative stress and accumulation of acetic acid is mechanistically associated with chronological aging (Burhans and Weinberger, 2009). Because morin could increase the longevity of stationary phase budding yeast culture (Figure 4), we question how it might play a role in modulating the growth and stress signalling pathways of yeast cells at the molecular level. In *S. cerevisiae* cells, the antioxidant defense includes enzymatic and non-enzymatic processes for scavenging ROSs to reduce cell toxicity. The lack of a cellular defense system results in increased accumulation of toxic radicals and cell death. Our results suggest that morin modulates the cell stress response via two different mechanisms, namely by reducing ROSs accumulation, as mentioned previously, and through transcriptional repression of ROS activation (Figures 3, 5). The latter may be due to its antioxidant property, which partially compensates for the requirement of antioxidant enzymes to get rid of toxic radicals. Further examination will be required to elucidate the mechanism of action of morin or other flavonoids to mitigate stress signalling in relation to aging. The mechanism might involve regulation of genes that encode antioxidant enzymes, among other actions. Many stress-responsive transcription factors modulate the stress response and ensure cell survival (Soontorngun, 2017). There are alterations in antioxidant enzymes as well as mitochondrial ribosomal and other proteins because acetic acid greatly disturbs mitochondrial functions, interrupts reserve metabolism, and interferes in central carbon metabolism and amino acid biosynthesis in yeast. Thus, not surprising, acetic acid has been identified as an aging accelerator as well as an inducer of programmed cell death (PCD) in several model organisms (Ludovico et al., 2001). Further investigation is one way to elucidate the interplay among these stress-responsive transcription factors and key signalling kinases linked to cell growth and aging. Nevertheless, in the search for anti-aging analogues of quercetin using a model yeast, among others morin appears to be an interesting candidate with potent antioxidant and anti-aging activities.

In fact, compelling evidence has shown that morin is a bioactive compound with multiple pharmacological and neuroprotective effects in which many cytokines and signalling pathways, including m*TOR*, contribute (Rajput et al., 2021). Increased bioavailability and better pharmacological actions of morin hydrate against numerous chronic and lifestyle-related degenerative diseases have been demonstrated (Rajput et al., 2021). Furthermore, in addition to functioning as antioxidants, some flavonoids also serve as metal-chelating agents by interacting with protein and lipid kinase signalling pathways, and others modulate mitogen-activated protein kinases, nuclear factor kappa B, and tyrosine kinases (Sunthonkun et al., 2019). Other flavonoids such as rutin, quercetin, fisetin, kaempferol, apigenin, myricetin, and glycitein possess anti-amyloidogenic and fibril-destabilization activities *in vitro* and act as metal chelators to suppress oxidative stress. Future work should focus on developing effective formulations of herbal drug or flavonoids that counteract Alzheimer’s disease and other chronic diseases and disorders and resolve the remaining challenges such as bioavailability. Recently, morin has been shown to counteract blood-brain barrier disruption and thus enhances the integrity of the blood-brain barrier against cerebral ischemia reperfusion in rats (Khamchai et al., 2020). These findings suggest that morin and other flavonoids could be used as natural dietary agents (Solairaja et al., 2021), natural chemotherapeutic drug (Caselli et al., 2016), or other versatile biological and pharmacological potential (Rajput et al., 2021).

In summary, aging is a conserved phenomenon between yeast and human, making yeast a simple but powerful tool to examine the anti-aging potential of bioactive substances. Here, some selected flavonoids, natural analogues of quercetin, were examined using *S. cerevisiae* as a model yeast to uncover the antioxidant and anti-aging properties and to establish their potential connection to cellular stress adaptation. Morin, kaempferol, aromadendrin, and steppogenin protected yeast cells against acetic-acid-induced oxidative stress, as shown by decreased cell sensitivity. There was also a reduction in intracellular ROSs following acetic acid treatment. Using the chronological aging assay, quercetin, morin, and steppogenin could extend the lifespan of wild-type *S. cerevisiae* by 15%–25%. Consistent with the fact that oxidative stress is a key factor in aging, acetic acid resistance was associated with increased expression of the gene *TOR1*, which encodes a key growth signalling kinase, and *MSN2* and *MSN4*, which encode stress-responsive transcription factors (Figure 5). Thus, yeast could be used as a versatile model organism to further study cross-talk between aging and cell signalling pathways.

Using *S. cerevisiae* as a model yeast, quercetin analogues extended the chronological lifespan through the antioxidative mechanism via activation of stress response signals. The detailed molecular mechanism behind their anti-aging prevention remains to be fully elucidated. Morin and other quercetin analogues appear to be promising candidates to promote better health and well-being. Thus, a yeast model system could be employed to facilitate the discovery of functional bioactive compounds and to assess the functional property and quality of bioactive compounds isolated from herbal extracts prior to undertaking animal or cell experiments that aim toward commercial product development.

## Data Availability

The raw data supporting the conclusion of this article will be made available by the authors, without undue reservation.

## Abbreviations

Δ, Deletion strain; ^0^C, Celsius; AA, Acetic acid; CLS, Chronological Lifespan; DCFH-DA, Dichloro-dihydro-fluorescein diacetate; DNA, Deoxyribonucleic acid; Hr, Hour; H_2_O_2_, Hydrogen peroxide; OD, Optical Density; Q, Quercetin; RLS, Replicative Lifespan; ROS, Reactive Oxygen Species; SODs, Superoxide Dismutase; *S. cerevisiae*, *Saccharomyces cerevisiae*; WT, Wild-type.
